# Mucosal Vaccination with Live Attenuated *Bordetella bronchiseptica* Protects against Challenge in Wistar Rats

**DOI:** 10.3390/vaccines11050982

**Published:** 2023-05-15

**Authors:** Beatriz Miguelena Chamorro, Karelle De Luca, Gokul Swaminathan, Nicolas Rochereau, Jade Majorel, Hervé Poulet, Blandine Chanut, Lauriane Piney, Egbert Mundt, Stéphane Paul

**Affiliations:** 1 CIRI—Centre International de Recherche en Infectiologie, Team GIMAP (Saint-Etienne), Université Claude Bernard Lyon 1, Inserm, U1111, CNRS, UMR5308, ENS Lyon, UJM, F69007 Lyon, France; beatriz_miguelena.chamorro.ext@boehringer-ingelheim.com (B.M.C.); nicolas.rochereau@univ-st-etienne.fr (N.R.); jade.majorel@univ-st-etienne.fr (J.M.); blandine.chanut@univ-st-etienne.fr (B.C.); 2 Boehringer Ingelheim, Global Innovation, F69800 Saint Priest, France; karelle.de_luca@boehringer-ingelheim.com (K.D.L.); gokul.swaminathan@boehringer-ingelheim.com (G.S.); herve.poulet@boehringer-ingelheim.com (H.P.); lauriane.piney@boehringer-ingelheim.com (L.P.); egbert.mundt@boehringer-ingelheim.com (E.M.); 3 3CIC Inserm 1408 Vaccinology, F42023 Saint-Etienne, France

**Keywords:** *Bordetella bronchiseptica*, live attenuated vaccines, rat model, mucosal vaccination, canine infectious respiratory disease complex (CIRDC)

## Abstract

*Bordetella bronchiseptica* (Bb) is a Gram-negative bacterium responsible for canine infectious respiratory disease complex (CIRDC). Several vaccines targeting this pathogen are currently licensed for use in dogs, but their mechanism of action and the correlates of protection are not fully understood. To investigate this, we used a rat model to examine the immune responses induced and the protection conferred by a canine mucosal vaccine after challenge. Wistar rats were vaccinated orally or intranasally on D0 and D21 with a live attenuated Bb vaccine strain. At D35, the rats of all groups were inoculated with 10^3^ CFU of a pathogenic strain of *B. bronchiseptica*. Animals vaccinated via either the intranasal or the oral route had Bb-specific IgG and IgM in their serum and Bb-specific IgA in nasal lavages. Bacterial load in the trachea, lung, and nasal lavages was lower in vaccinated animals than in non-vaccinated control animals. Interestingly, coughing improved in the group vaccinated intranasally, but not in the orally vaccinated or control group. These results suggest that mucosal vaccination can induce mucosal immune responses and provide protection against a Bb challenge. This study also highlights the advantages of a rat model as a tool for studying candidate vaccines and routes of administration for dogs.

## 1. Introduction

*Bordetella bronchiseptica* (Bb) is a Gram-negative coccobacillus of the genus Bordetella, which encompasses 16 other species, including *Bordetella pertussis*. *B. bronchiseptica*, can infect a wide range of mammals, including dogs, cats, rodents, and pigs [1]. In dogs, Bb is the major causative agent of canine infectious respiratory disease complex (CIRDC) or kennel cough, while other pathogens such as *Mycoplasma cynos* and canine parainfluenza virus (CPIV) are also frequently detected [2]. Nevertheless, Bb was frequently identified as the causal agent of CIRDC, as seen in 3.3% to 78.7% of canine samples in Europe [3]. CIRDC is characterized by tracheobronchitis, manifesting as a cough, lethargy, and intranasal discharge, and on rare occasions, it can lead to [4] or lethal bronchopneumonia in puppies [5]. Moreover, transmission to the owner is possible [6,7]. Bb has also been implicated in respiratory infections in immunocompromised humans [8], and occasionally even in immunocompetent humans [9]. In dogs, the current standard of care is based on treatment with antibiotics and supportive care for clinical signs. However, the attachment of the bacteria to the cilia may necessitate the nebulization of the antibiotics [10]. Vaccines are an essential tool for the protection of animals and humans against this pathogen.

Several vaccines with various routes of administration (oral, intranasal, or injectable) are currently licensed for clinical use in dogs [11]. The mucosal vaccines contain live attenuated strains, whereas the injectable vaccines consist of inactivated bacteria or antigen extracts [11]. A number of studies have compared the efficacy of the vaccines licensed for dogs and the immune responses they induce [12,13,14,15,16]. The parameters studied are generally limited to the observation of clinical signs (cough, fever, sneezing, and lethargy), colonization and bacterial shedding, and the induction of hemagglutinating antibodies in the serum. However, some studies have also considered mucosal immune responses, such as the induction of Bp-specific IgA [15]. Mucosal vaccination against Bb via the oral or intranasal route has been reported to lead to better protection than the use of injectable vaccines does [15,16]. Nevertheless, it is not possible to conclude that the route of administration is solely responsible for these observed differences, because the formulation of the vaccines used also differs. However, in comparison to vaccination via the intranasal and oral routes, the superiority of one route over the other remains a matter of debate [14]. The intranasal route more closely resembles the natural route of infection, but oral vaccines are easier to administer and can also induce mucosal immune responses. Notably, Larson et al. showed that intranasal and oral vaccines were similarly effective against Bb in dogs, both being superior to the inactivated parenteral vaccine [16]. Another study comparing oral and intranasal vaccination reported no significant differences in protection following vaccination via these two routes [12]. Moreover, vaccination via either of these routes can lead to more than 12 months of protection in dogs [17,18].

The main weakness of all the studies cited above is their lack of consideration of cellular responses, the colonization of other organs and the induction of immunological memory in the vaccinated animals. There is a critical need to develop a model that can enable an improved understanding of the mechanisms underlying the efficacy of each vaccine. This should facilitate the future development of new vaccines in dogs, for which an understanding of the best way to efficiently induce mucosal immune responses will be key. Novel candidates targeting both *B. bronchiseptica* and *B. pertussis*, thereby protecting both humans and animals, have been studied [19,20]. A relevant model for preclinical validation is required for the evaluation of these vaccines. Interestingly, the infection of rats with these bacterial species reproduces the lead clinical sign of CIRDC in dogs, the cough [21]. For this reason, the rat model has been used to study the pathogenesis of Bordetella infections and responses to vaccination against these pathogens for more than a century [21,22,23,24,25].

In this study, we used a rat model to investigate and compare established vaccine strains against a *B. bronchiseptica* challenge infection dosed through various routes. We previously studied the pathogenesis and induction of disease in Wistar rats after challenge with Bb, as previously described [21]. Here, a live attenuated vaccine strain was used. The vaccine strain was administered orally or intranasally for immunization of the animals, which were subsequently challenged. Numerous end points, including, cough, colonization, lesions, and humoral and cellular immune responses between groups were evaluated. Importantly, we observed that vaccine administration via either the oral or intranasal route prevented colonization and induced specific local and systemic antibody responses. However, cellular immune responses in the spleen and clinical signs differed between routes of administration. Overall, the observed data suggest that mucosal vaccination effectively protects rats against a Bb challenge infection. Such detailed understanding of the specific immunological and clinical outcomes influenced by mucosal routes of vaccination in translatable animal models have important implications in the field of vaccinology and infectious disease research.

## 2. Materials and Methods

### 2.1. Vaccine Strain, Growth, and Vaccination

The live attenuated strain of Bb present in the oral Recombitek vaccine [17] was used for vaccination. The vaccine strain was grown in TSB-ST (BioMérieux SA, Marcy l’étoile, France) at 37 °C, with shaking at 180 rpm, for 14 h. The bacterium was cultured until an OD_600_ of 2.5 (approximate equivalent to 1.5 × 10^9^ CFU/mL) was reached. At D0 and D21, female Wistar rats (3–4 weeks old) were anesthetized with isoflurane for nasal administration. The live attenuated vaccine strain was then administered intranasally (10^6^ CFU/animal) or orally (10^8^ CFU/animal) with a micropipette. The bacterium was diluted in phosphate-buffered saline (PBS); the inoculation volume used was 50 µL/animal for intranasal administration (25 µL in each nostril), and 100 µL for oral micropipette-guided drug administration (MDA) [26]. One group of animals received PBS under the same conditions, as a non-vaccinated control.

### 2.2. B. bronchiseptica Strain, Growth, and Challenge

A virulent Bb strain of canine origin was used for challenge. This challenge strain was grown in TSB-ST (BioMérieux SA, Marcy l’étoile, France) at 37 °C, with shaking at 180 rpm, for 6 h. The bacterial culture was collected when an OD_600_ of 1 was reached (approximately equivalent to 1 × 10^9^ colony-forming units (CFU)/mL). On D35, all the study animals were anesthetized with isoflurane. Rats were inoculated with 25 µL of the challenge strain in each nostril, resulting in a total dose of 50 µL of the strain in PBS, or a titer of 1 × 10^3^ CFU/animal. The diluted bacterial cultures were back-titrated to confirm the titers used (CFU/animal). Each suspension was serially diluted and 10 µL drops were dispensed onto Bordet Gengou agar containing 15% sheep blood (Becton Dickinson, Heidelberg, Germany). 

### 2.3. Vaccine-Specific Antibody Detection via ELISA

Antibody titers were determined using an enzyme-linked immunosorbent assay (ELISA) to evaluate the levels of Bb-specific antibodies in the serum, intranasal washes, and feces of vaccinated and challenged rats. Blood was sampled via the intracardiac route into BD Microtainer SST Tube (Becton Dickinson, Heidelberg, Germany) and incubated for 30 min at room temperature. All blood samples were centrifuged at 10,000× *g* for 10 min at 4 °C. The supernatants were harvested and stored at −20 °C until analysis. Feces were added to an Eppendorf tube, with 1 mL of PBS and a 1% 1× protease inhibitor cocktail (Thermo Scientific, Rockford, IL, USA) per 100 mg of the sample, and shaken until a homogeneous suspension was obtained. Intranasal washes were sampled after the animals had been euthanized, by flushing 400 µL of PBS through the trachea and collecting it via the nares. Fifty microliters of each intranasal sample was added to a DNase- and RNase-free tube, which was then stored at −80 °C until analysis. We added 35 µL of a 10× protease inhibitor cocktail to the remaining volume. Fecal and intranasal samples were centrifuged at 10,000× *g* for 10 min at 4 °C, and the supernatants were harvested and stored at −20 °C until analysis.

Bb vaccine strain-specific ELISA plates were coated with 50 µL/well of a 5 µg/mL suspension of the sonicated live attenuated Bb strain. The coated plates were incubated overnight at 4 °C, washed three times with 200 µL per well of 1× PBS-0.05% Tween 20 and blocked via incubation with 1× PBS- 5% BSA (5% bovine serum albumin in PBS) for 1 h at 37 °C. The plates were washed again as described above, and the sera was diluted in a range of 1:1000 to 1:20,000 in 1× PBS for sera, while for intranasal washes and feces, a dilution of 1:2 to 1:4 was used. Fifty microliters of the resulting solutions were added per well. The plates were incubated for 2 h at 37 °C and then washed three times with 200 µL of 1× PBS-0,05% Tween 20. They were incubated with 50 µL/well of anti-rat IgG and IgM (1:6000 dilution) and IgA (1:10,000 dilution) in 1× PBS-BSA 5% (Bio-Rad Laboratories, Inc., Hercules, CA, USA) for detection. The plates were incubated for 1 h at 37 °C and washed three times, as previously described. In the next step 50 µL of 3,3′, 5,5”-tetramethylbenzidine (TMB)/well was added and the plates were incubated for 15 min in the dark at room temperature. The reaction was stopped by adding 50 µL of hydrochloric acid (HCl)/well. The titers of rat IgG, IgM, and IgA antibodies were determined on a TECAN microplate reader (Tecan Austria GmbH, Grödig, Austria), via a colorimetric evaluation of the stopped reactions on the ELISA plate at a wavelength of 450 nm.

### 2.4. Detection Challenge and Vaccine Strain

Bacterial load was determined via specific quantitative real time polymerase chain reactions (qPCR) and culture on blood agar plates. On D-1, 13 days post-boost and 7 and 14 days post-challenge (p.c.), 4 rats per group were euthanized with pentobarbital and their tracheas and lungs were removed, weighed, and minced in 5 mL of Dulbecco phosphate-buffered saline (D-PBS). Samples were homogenized with a syringe piston and passed through a cell strainer with 70 µm pores. The cell strainer was washed with D-PBS, and the suspension was collected and centrifuged at 6000× *g* for 5 min at 22 °C. At this step, 200 µL of each sample was added to DNase- and RNase-free tubes for qPCR and frozen at −80 °C until analysis. The tissue suspension obtained was serially diluted in D-PBS and plated on blood agar plates. The bacteria were cultured at 37 °C for 2 days, and the number of CFU was determined. The number of viable colonies were counted manually, and the presence/absence of hemolysis was used to differentiate visually between the vaccine (non-hemolytic) and challenge (hemolytic) strains (Supplementary Materials, Figure S1). The identity of the colonies corresponding to Bb was confirmed by mass spectrometry using MALDI-TOF (BRUKER, Billerica, MA, USA).

Analysis was performed to differentiate between the Bb-challenge or Bb-vaccine strain, employing strain specific qPCR using primers specifically targeting sequences of either the Bb-challenge or the Bb -vaccine strain. Frozen tracheal and lung homogenates and nasal lavages were thawed. DNA was extracted with DNeasy Blood and Tissue Kit (QIAGEN GmbH, Hilden, Germany) in accordance with the manufacturer’s protocol. DNA quantity and quality were assessed with NanoDrop 2000c (Thermo Fisher Scientific, Waltham, MA, USA). Reactions were then set up, in duplicate, on a 96-well qPCR plate. The reaction mixture consisted of 5 µL of the DNA-containing samples, 12.5 µL of 1× Master Mix Quantitect Multiplex PCR (QIAGEN GmbH, Hilden, Germany), 0.5 µL of each primer (listed in Table 1), and 0.25 µL of a probe and RNAse-free water to obtain the total volume of 20 µL. The reaction mixture was subjected to an initial denaturation step at 95 °C for 15 min, followed by 40 cycles of 94 °C for 60 s, and then 60 °C (vaccine strain) or 64.5 °C (challenge strain) for 60 s.

### 2.5. Analysis of Cough

The clinical signs of three animals per group were monitored at three different timepoints: 6, 9, and 12 days post-challenge. Each animal was filmed separately, for 10 min, with 4K Video Camera VX980 (Panasonic, Osaka, Japan) and the noise was monitored with an MKE 600 microphone (Sennheiser, Wedemark, Germany). The number of coughs was analyzed with Adobe Premier Pro (Adobe Systems Inc., San José, CA, USA), based on the recorded images and sound waveforms.

### 2.6. Histology Trachea and Lung

For the histological analysis, 2 cm of each lung and trachea was dissected after challenge and added them to 3 mL of 1× PBS supplemented with 4% paraformaldehyde (PFA) (SIGMA-ALDRICH, St. Louis, MO, USA). These samples were sent to Vet Diagnostics (VetDiag, Lyon, France) for microscopical analysis. Sliced samples were stained with hematoxylin-eosin (H-E) in accordance with standard procedures. Microscopic lesions and/or the presence of bacteria were analyzed under a Nikon Eclipse Ci light microscope (Nikon Europe B.V.). The analysis was performed by an external certified veterinary pathologist who had no knowledge of the group allocation. The scoring system used included categories such as absence of lesions, minimal, mild, or moderate, based on the level of inflammation characterized by cellular infiltration, epithelial alteration, degeneration, and the presence of lymphoid follicles.

### 2.7. Isolation of Splenocytes and Flow Cytometry Analysis

Spleens were harvested before challenge, 7 and 14 days post-challenge. They were homogenized mechanically and passed through a Nylon Corning^®^ cell strainer with 100 µm pores (Corning Incorporated, Durham, NC, USA). The cell suspension was layered into Ficoll-Paque™ PREMIUM (Cytiva, Uppsala, Sweden) and centrifuged at 400× *g* at 10 min at 20 °C without the brake on. Cells were harvested and washed twice with 1× PBS. Part of the splenocytes were stained with trypan blue and counted by eye under a microscope (XYZ). The cells were frozen in a solution containing 50% RPMI, 40% fetal bovine serum (FBS) (Eurobio scientific, Les Ulis, France), and 10% dimethyl sulfoxide (DMSO) (SIGMA-ALDRICH, St. Louis, MO, USA) and stored in liquid nitrogen until analysis.

The cells were thawed in a 37 °C water bath, transferred to tubes containing a medium supplemented with 10% RPMI, 5% FBS, and 1× Antibiotic Antimycotic Solution (SIGMA-ALDRICH, St. Louis, MO, USA) and centrifuged at 400× *g* for 10 min at 20 °C. Splenocytes were counted, viability was assessed after trypan blue staining, and cells were washed twice as described above. The cells were then activated via incubation with Cell Activation Cocktail (with Brefeldin A) (BioLegend, San Diego, CA, USA) for 5.5 h at 37 °C. The activated splenocytes were stained for viability and incubated for 25 min in the dark. The cells were then washed twice as described above, and surface-stained via incubation with the antibodies indicated in Table 2 for 20 min. Cells were washed as described above and fixed via incubation with a 1× FOXP3 Fix/Perm solution (Thermo Fisher Scientific, Waltham, MA, USA) for 30 min in the dark. Samples were washed twice with Permeabilization Buffer (Thermo Fisher Scientific, Waltham, MA, USA) and incubated with the indicated diluted intracellular antibodies for 30 min. The cells were then washed twice as described above with Permeabilization Buffer and fixed with 1% paraformaldehyde diluted in 1× PBS. The flow cytometry data obtained were analyzed with FlowJo version 10.8.1 (FlowJo, Becton, Dickinson and Company, NJ, USA).

### 2.8. Statistics

Statistical analyses were performed with GraphPad Prism version 9 (GraphPad, San Diego, CA, USA). Differences between groups were evaluated using one-way ANOVA with Tukey tests for multiple comparisons and using two-way ANOVA for the comparison of multiple variables. There were three biological replicates for the cough analysis, and four replicates for all other analyses. Correlograms were generated with R Studio Software Version 4.0.2 (RStudio, Boston, MA, USA), and the multiple R-squared was calculated for each group of variables.

### 2.9. Animal Care and Use

All rats were housed in identical conditions at the university hospital unit for animal testing (Saint-Etienne, France). The experimental protocol was designed in accordance with French law (decree number 2001-464 29/05/01) and the recommendations of the European Economic Community (86/609/CEE) for the care and use of laboratory animals (permit no. #23498-2020010715103375).

## 3. Results

### 3.1. Mucosal Vaccination against B. bronchiseptica Elicits Robust Systemic and Mucosal Humoral Responses

**Figure 1:**
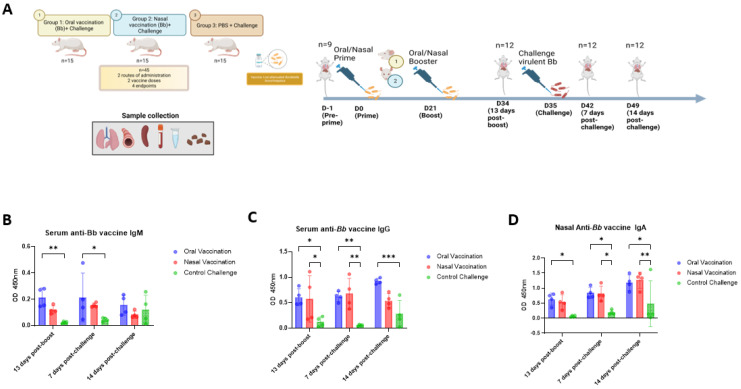
Study design and humoral responses after vaccination and challenge. Study design of vaccination and challenge model of Wistar rats. Animals were vaccinated intranasally or orally at D0 and D21 and challenged at D35 (**A**). Intranasal and oral vaccination induced systemic and mucosal anti-*B. bronchiseptica* vaccine IgG, IgM, and IgA. Blood was collected 13 days post-boost, and 7- and 14-days post challenge upon euthanasia via the intracardiac route. Feces and nasal lavages were collected at the same timepoint. Anti-Bb vaccine IgG and IgM were measured in serum and differences in the OD were compared among groups and timepoints (**B,C**). Anti-Bb vaccine IgA was measured in nasal lavages and OD was compared between groups and timepoints (**D**). The results are shown with the OD and mean with SDs are presented. (n = 4 per group per day). *p* values were determined using two-way ANOVA (* *p* ≤ 0.05; ** *p* ≤ 0.01; *** *p* ≤ 0.001).

We included 45 4-week-old Wistar rats divided in 3 groups for investigations of the immunogenicity and protection of mucosal vaccination against Bb. The rats in the two treatment groups were vaccinated with the Bb live-attenuated vaccine strain, via the oral route in one group and intranasally in the second group. The third group of rats was left unvaccinated and served as a challenge control. All groups were challenged 35 days after vaccination. Three or four animals per group were euthanized at each of the four time points, and blood, feces, intranasal washes, the trachea, lungs, and spleen of each animal were obtained for analysis (experimental set-up outlined in Figure 1A).

Protection against mucosal pathogens might require the induction of both systemic and mucosal responses at local sites [27,28]. It was hypothesized that mucosal vaccine administration would induce potent mucosal responses inducing protection against colonization. To this end, a Bb-specific ELISA was used to investigate the development of mucosal and systemic antibodies before challenge and 7 and 14 days p.c. Bb-specific IgA antibodies were determined in serum, nasal lavages, and feces, whereas Bb-specific IgG and IgM were determined in serum only. Before challenge infection, Bb-specific IgM titers were higher in the oral group than in the intranasal and control groups (Figure 1B). Bb-specific IgG titers in serum and Bb-specific IgA titers in intranasal lavage were similar between the two vaccinated groups, and significantly higher in these two groups than in the control group (Figure 1C,D). The Bb-specific IgM titers of the vaccinated groups remained steady after challenge, until day 14 p.c., when they started to decrease (Figure 1B). This decline is consistent with the expected dynamics of the humoral immune responses, in which IgM antibodies are the first to appear, before class-switching, which usually results in an increase in the affinity of the antibodies synthesized in the B cells [29]. The control group showed a slight increase in Bb-specific IgM titers after challenge infection, which is consistent with what is expected from a first encounter with the pathogen (Figure 1B). Bb-specific IgG titers remained constant 7 days p.c. in the vaccinated groups and were significantly higher in vaccinated animals than in the control group (Figure 1C). However, on day 14 p.c., an increase in Bb-specific IgG titers was observed in the oral group, but not in the intranasal group (Figure 1C), resulting in a significant superiority of the immune response after oral vaccination over the control group, but no such superiority after vaccination via the intranasal route (Figure 1C). Finally, the data show that the Bb-specific IgA responses revealed a slight increase in these response in the nasal lavages of both vaccinated groups after challenge infection (Figure 1D). On day 14 p.c., the titers of Bb-specific IgA in both groups of vaccinated rats had significantly increased and were slightly higher for the intranasal group when compared with those for the orally vaccinated group, relative to the control group (Figure 1D). Additionally, the binding strength (avidity) of Bb-specific IgA in vaccinated animals was higher in intranasal than orally vaccinated animals (Supplementary Materials, Figure S2). Besides, minimal or no Bb-specific IgA responses were detected in the feces and serum, both before or after challenge (Supplementary Materials, Figure S3). Together, these results demonstrate that both the oral and intranasal routes of vaccination can induce Bb-specific systemic and mucosal antibodies, both after vaccination and after challenge with the challenge strain.

### 3.2. Mucosal Vaccination Prevents Challenge Strain Colonization of the Respiratory Tract during Replication of the Vaccine Strain

**Figure 2:**
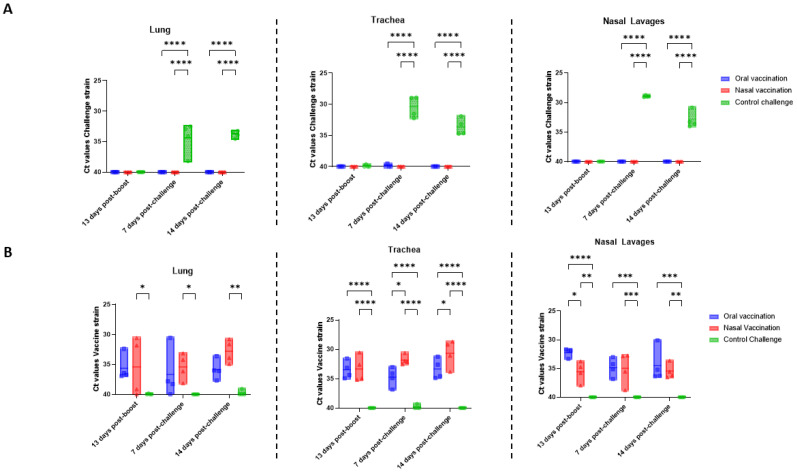
Intranasal and oral administration reduces bacterial burden in lung, trachea and nasal lavages after challenge and vaccine strain persist in the respiratory tract up to 28 days after boost. Briefly, 13 days after the boost and 7 and 14 days after challenge, the presence of challenge and vaccine strain in the lung, trachea and nasal lavages of euthanatized rats was determined via qPCR. Challenge strain was only detected in the unvaccinated rats 7 and 14 days after challenge in the lung, trachea, and nasal lavages (**A**). The vaccine strain was detected in the vaccinated animals and compared between different days in each organ (**B**). Ct values were obtained via real-time qPCR analysis. Each dot represents an individual rat. The error bars represent the standard error of the mean (n = 4 per group). The asterisks and brackets refer to statistical significance determined via ANOVA with multiple comparisons. (* *p* ≤ 0.05; ** *p* ≤ 0.01; *** *p* ≤ 0.001; **** *p* ≤ 0.0001).

The mucosal administration of live attenuated Bb has been shown to prevent colonization in dogs [17,18]. We investigated whether or not the same vaccination procedure in rats also decreased Bb load in the respiratory tract of this species. Moreover, the standard detection method for Bb in dogs is the culture of bacteria from intranasal swabs. Here, the detection of bacteria in lavages from three organs was explored: the trachea, lung, and nose. A quantitative real time PCR (qPCR) was employed to differentiate between the vaccine and challenge strains. Tracheal, lung, and nasal lavages from the animals were sampled 13 days post-boost and 7 and 14 days p.c. Samples were processed and analyzed via qPCR with primers specifically targeting either the challenge or the vaccine strain. The Ct values resulting from the real-time qPCR were studied and compared between animals (hereafter referred to as Ct values). Before vaccination and challenge infection, the absence of the bacterium from the trachea, lung, and nasal lavages of nine animals was tested; and neither the vaccine strain nor the challenge strain was detected (data not shown). Bacterial load in the upper and lower respiratory tract decreased significantly, at 7 and 14 p.c., in animals vaccinated orally or intranasally, where the challenge strain was not detected (Figure 2A). In contrast, the challenge strain was present at significant levels, at all three sites studied, after challenge in the non-vaccinated control (Figure 2A). Bacterial loads in the lungs were low but were similar at 7 and 14 p.c. in the control group (Figure 2A). Also, in the control group, the bacterial loads in the trachea and nasal lavages were initially higher but were significantly reduced 14 days post-challenge (Supplementary Materials, Figure S4). The data demonstrate that the challenge strain of Bb effectively colonizes non-vaccinated animals, and that mucosal vaccination prevents this phenotype.

Post-vaccination, the presence and localization of the vaccine strain was also investigated. Reassuringly, the vaccine strain was not detected in the control group (Figure 2B). Expectedly, the vaccine strain was present in all vaccinated animals, for up to 28 days after the second vaccination (Figure 2B). Animals vaccinated orally or intranasally had similar levels of the vaccine strain in the lung until 14 days p.c., after which, levels were higher in the intranasally vaccinated animals compared to those in the orally vaccinated animals (Figure 2B). Similarly, investigations of vaccine strain levels in the trachea revealed similar levels in the two vaccinated groups before challenge infection, with a significant increase in the intranasally vaccinated, but not orally vaccinated animals after challenge (Figure 2B). Finally, vaccine strain levels in intranasal lavages were higher in orally vaccinated animals than in intranasally vaccinated animals before challenge infection (Figure 2B). After challenge, vaccine strain levels increased in intranasally vaccinated rats, but, by 14 days p.c., vaccine strain levels were again superior within the orally vaccinated group (Figure 2B). Overall, oral, or intranasal immunization led to a decrease in bacterial load in the nose, trachea, and lungs, 7 and 14 days p.c., as seen from the lack of detection of the challenge strain. This decrease may be associated with the persistence of the vaccine strain within the upper and lower respiratory tracts, following vaccination via either of the routes.

### 3.3. Bb-Specific Antibodies Show Strong Negative Correlation with Bacterial Burden While Vaccine Persistence Is Weakly Associated with Antibody Titers

**Figure 3:**
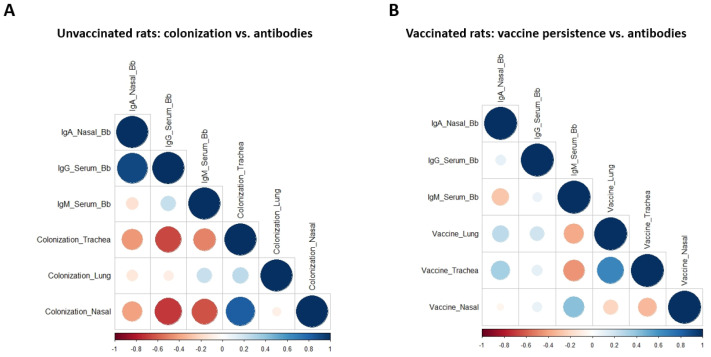
Mucosal and systemic antibodies correlate with lower bacterial burden in the respiratory tract. Correlograms values were calculated with R. Unvaccinated rats 7 and 14 days after challenge showing a strong negative correlation between the colonization and the antibodies (**A**). Antibodies of vaccinated animals which were positive but not strongly correlated with the vaccine presence in the respiratory tract (**B**). Positive correlations appear as blue circles and the negative correlations appear from orange to red. The size of the circle indicates the strength of the correlation.

In the next step, we investigated the correlation between the presence of the vaccine strain in the respiratory tract and the induction of both mucosal and systemic antibodies. In addition, it was also investigated whether the presence or absence of these antibodies was correlated with the bacterial load in the control group after challenge. Correlograms were generated to reveal either positive or negative correlations between the various parameters. The R2 value is close to 0 if there is no correlation observed [30]. If two variables are positively correlated, they both increase together or decrease together (similar patterns of change). A negative correlation implies that, as one variable increases, the other decreases (opposite patterns of change). Bb-specific IgA levels in the nose and Bb-specific IgG levels in the serum were strongly negatively correlated with colonization of the trachea (R2 = 0.91) and nose (R2 = 0.96) following challenge infection (Figure 3A). Thus, higher levels of antibodies in the serum or nasal cavity were associated with a lower bacterial load in the respiratory tract. Moreover, the presence of Bb-specific antibodies in serum (IgG) was positively correlated with the detection of IgA in the nose (R2 = 0.82) (Figure 3A). Conversely, the presence of Bb-specific IgA in the nose was positively correlated with the presence of the vaccine strain in the trachea and lung, although this correlation was weak (R2 = 0.118) (Figure 3B).

### 3.4. Mucosal Vaccination Protects against Cough in B. bronchiseptica-Infected Wistar Rats

**Figure 4:**
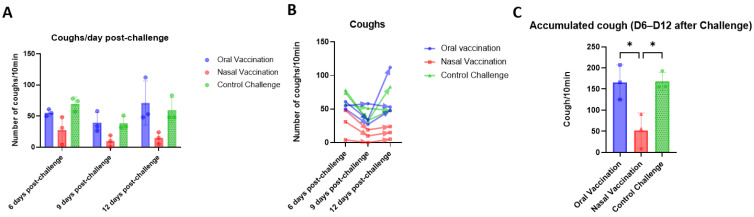
Intranasal vaccination of live-attenuated *B. bronchiseptica* vaccine decreases cough in infected rats. Coughs were counted and recorded in three animals per groups and three times after challenge (6-, 9- and 12 days p.c.), for 10 min each. Represents the number of coughs per day per each group and the mean per group and day were compared between each other (**A**). The number of coughs per animal are presented day by day (B). The accumulated cough of the three days was summed up and compared between groups (**C**). *p* values were determined by two-way ANOVA (* *p* ≤ 0.05).

Furthermore, it was investigated if vaccination provides protection against the main clinical sign of CIRDC, cough. It was hypothesized that the induction of mucosal and systemic Bb-specific antibodies observed with both routes of administration would attenuate clinical signs. It was shown before that mucosal vaccination against *Bordetella pertussis* attenuated coughing in challenged rats [22]. In this study, rats were vaccinated on day 0 and day 21, with 10^8^ CFU orally and 10^6^ CFU intranasally, and then challenged on day 35 with a virulent Bb strain. Video recordings were performed on three animals from each group on days 6, 9, and 12 p.c. and subsequently analyzed. On day 6, control animals coughed a mean of 70 times in 10 min, whereas orally vaccinated rats coughed a mean of 50 times while intranasally vaccinated rats coughed a mean of 30 times. Thus, on day 6, coughing was reduced in both vaccinated groups, but this difference with respect to the non-vaccinated controls was significant only for the intranasal group (Figure 4A). On day 9, coughing had decreased in all groups and there were no significant differences between any of the groups. Finally, 12 days after challenge infection, coughing increased in the orally vaccinated and control rats, to levels significantly higher than those in intranasally vaccinated rats, which remained low (Figure 4A,B). Observations of the cumulative numbers of coughs over the three days of recording revealed significantly fewer coughs in the intranasally vaccinated animals than in the orally vaccinated and not vaccinated control animals, with similar numbers of coughs in these last two groups (Figure 4C). These data suggest that intranasal vaccination reduces cough caused by virulent Bb strains, whereas oral vaccination does not.

### 3.5. Microscopical Analyses of the Trachea and Lung

Given the differences in clinical signs between groups of animals with apparently similar immune responses and protection, microscopic analyses were performed on the respiratory tract. It was investigated whether or not the presence of cell infiltrates and inflammation in the lung and trachea could account for the differences in clinical signs. The lungs and trachea were collected from animals at 7 and 14 days p.c., formalin fixed, and H&E-stained. In the trachea, 7 days p.c., minimal diffuse mononuclear inflammation lesions were observed in all vaccinated animals, but epithelial alterations were observed only in the intranasally vaccinated group (Supplementary Materials, Table S1). The epithelial alterations were characterized by minimal degeneration and were multifocal.

**Figure 5:**
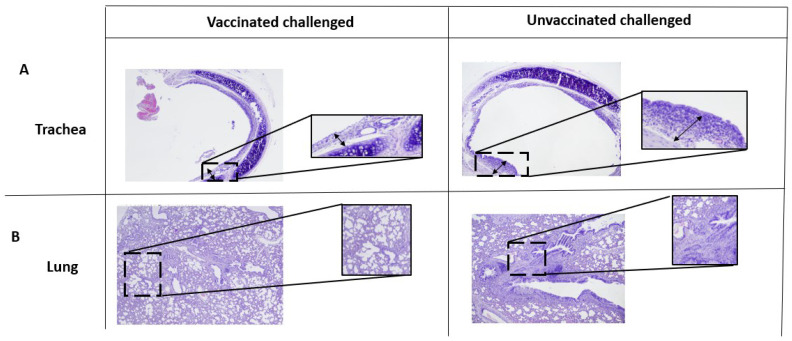
Signs of inflammation in the lung and trachea after challenge. Lung and trachea of each animal after challenge were stained with hematoxylin and eosin (H&E) and images are displayed at 20× magnification. Lesions of the trachea of vaccinated and unvaccinated animals after challenge are shown (**A**). Lesions of the lung of vaccinated and unvaccinated animals after challenge are shown (**B**). Four animals per group.

However, 14 days p.c., no lesions were observed within the trachea of orally vaccinated rats and only one intranasally vaccinated rat presented minimal inflammation of the trachea. In contrast, the unvaccinated rats presented minimal-to-mild mononuclear inflammation, and one of these animals also presented epithelial alterations (Figure 5A).

However, observations of the lung 7 days p.c. revealed similar bronchoalveolar inflammation in all groups. Interestingly, lymphoid follicle formation was visible in one animal per group of vaccinated rats, but not in the control. In the later stages, 14 days after challenge, the lesions were more severe, particularly in the control group. For instance, bronchoalveolar inflammation was common, but inflammation of the bronchus and lymphoid follicles appeared to be more severe in the control group (Figure 5B). Thus, mucosal vaccination reduced respiratory tract inflammation and lesions two weeks after challenge, whereas no such decrease was observed in the control group (Supplementary Materials, Figure S5).

### 3.6. Vaccination via the Oral and Intranasal Routes Elicits Different Proportions of Th17 and Treg Cells in the Spleen

Given the inflammation observed in the lung and trachea of both vaccinated and control animals, we investigated the cell populations present systemically, in the spleen, as a means of deciphering the mechanisms involved. A Th17-type immune response was described as necessary to fight *Bordetella* species [20]. However, the induction of a balanced immune response at the mucosal and systemic levels is essential, and an excessive inflammatory response can increase the severity of the disease, as also observed for SARS-CoV-2 infection [31].

**Figure 6:**
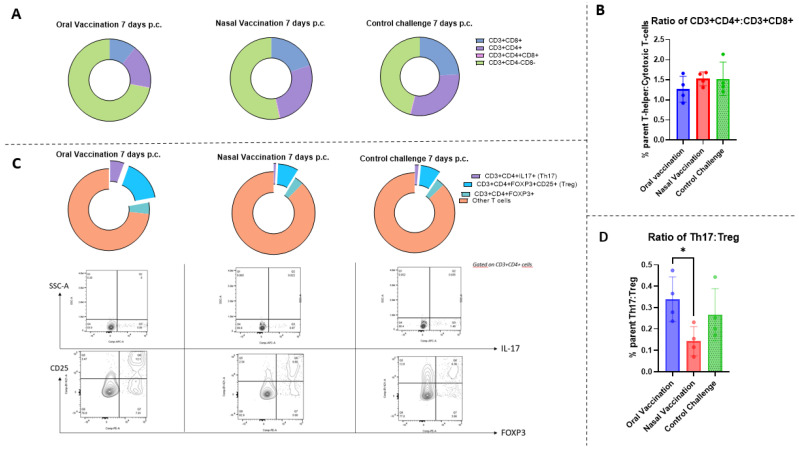
Oral vaccination leads to increase in Th17:Treg ratio of cell within splenic T-cells 7 days after challenge. Percentage of parent of CD3+CD4+, CD3+CD8+ and double-negative and doble-positive T-cells (**A**). Ratio of the percentage of CD4+ cells to the percentage of CD8+ cells within the CD3+ population (**B**). Percentage of parent and dot plots of CD3+CD4+IL17+ (Th17) and CD3+CD4+FOXP3+CD25+ (Treg cells) (**C**). Ratio of the percentage of parent of CD3+CD4+IL17+ to the percentage of parent of CD3+CD4+FOXP3+CD25+ (**D**). Each dot represents an individual rat. The error bars represent the standard error of the mean (n = 4 per group). The asterisks and brackets refer to statistical significance determined via ANOVA with multiple comparisons; * *p* ≤ 0.05.

It was hypothesized that one of the routes of administration might result in an unbalanced immune response. To analyze this, the ratio of Th17 (CD3+CD4+IL-17+), inflammatory T-cells, to Treg (CD3+CD4+ FOXP3+ CD25+), regulatory T-cells, was investigated (Supplementary Materials, Figures S6 and S7). The spleens were harvested, processed, and stained for flow cytometry on day 7 p.c. The percentage of both T-helper cells (CD3+CD4+) and cytotoxic T-cells (CD3+CD8+), were lower in the orally vaccinated rats than in the intranasally vaccinated or control animals (Figure 6A). However, the ratio of these two populations did not differ significantly between all groups (Figure 6B).

Investigations of T-cell subsets revealed that the proportions of Th17 and Treg cells were higher in the oral group than in the other groups (Figure 6C). Moreover, orally vaccinated rats had a significantly higher Th17:Treg cell ratio than intranasally vaccinated and control animals had (Figure 6D). Therefore, there were differences in immune cell populations in the spleen of animals vaccinated by different routes, and these differences were related to the route of administration.

## 4. Discussion

*B. bronchiseptica* is the main causal agent of CIRDC in dogs, and several vaccines against this bacterium are available on the market [11]. However, limited knowledge about the causality of the efficacy of these vaccines has been published. A bridging animal model in rats was investigated as a possible tool for exploring and studying the underlying mechanisms. To this end, the mucosal administration of a live attenuated *B. bronchiseptica* vaccine strain for dogs was investigated in Wistar rats. The over-arching goals for this study were (1) to explore the differences in the immune responses elicited via either oral or intranasal routes in rats and (2) to investigate aspects not previously described in dogs.

It was observed that vaccination via the oral and intranasal routes induced mucosal and systemic humoral responses while preventing bacterial colonization by a challenge strain and a lower number of lesions in the respiratory tract when compared with the non-vaccinated controls. However, intranasally vaccinated animals coughed significantly less frequently after challenge and had an altered ratio of inflammatory to regulatory cells in the spleen. Importantly, these findings are consistent with the immune response observed in dogs. Both oral and intranasal administrations of a *B. bronchiseptica* vaccine in dogs elicit Bb-specific antibodies in the serum [12,14,17,18,32]. A study comparing oral with intranasal vaccination in dogs reported no significant difference in median micro-agglutination titers (MAT) in the serum [14], which is consistent with our observations (Figure 1B,C). Antibody titers in the unvaccinated animals were heterogeneous, and lower than those in the vaccinated animals after challenge. This may reflect an immune evasion by and the persistence of *B. bronchiseptica*, leading to a defective immune response. *B. bronchiseptica* lacking the type III secretion system (T3SS) has been shown to elicit higher levels of Bp-specific antibodies [33,34]. It is likely that vaccination with a live attenuated strain will impede the modulation of the humoral response by virulent bacteria, while facilitating the recognition of all the immunostimulatory antigens for efficient antibody production [35].

*B. bronchiseptica* is a primary respiratory pathogen; thus, mucosal immune responses are likely crucial for protection. This has already been reported for *B. pertussis* [28]. Pathogen-specific IgA is the most important antibody response in mucosal tissues [27], and their detection serves as an indicator of protective mucosal immunity. In this study, it was observed that oral and intranasal vaccine administrations elicited comparable specific IgA responses in the nasal cavity (Figure 1D). However, the binding strength of nasal IgA and IgA levels in serum were higher in the intranasal group (Supplementary Materials, Figures S2 and S3). In dogs, intranasal administration results in a significant increase in IgA levels in the nose and faster induction of antibody production into the serum than subcutaneous administration does [15]. Another study comparing the oral, intranasal, and injectable routes of vaccination reported IgA titers in nasal swabs that were low before challenge, but that were higher in the orally vaccinated group than in the nasal and control groups [16]. Different observations were reported for an assessment of the same routes of administration of a vaccine against *B. pertussis* in rats [22]. Hall et al. investigated IgA levels in the lung and nose after challenge and found that only intranasal administration elicited IgA in the nose [22]. This discrepancy may reflect the disadvantages of the administration of the vaccine via oral gavage compared to the method of micropipette-guided drug administration (MDA), used here. MDA involves delivering the vaccine directly into the mouth with a micropipette, after training the animals to ensure correct intake [26]. This type of administration may lead to specific immune responses being elicited in the mouth or adjacent mucosae before arriving in the stomach, whereas gavage involves delivery directly to the stomach. MDA may constitute an alternative to gavage, more closely resembling administration in dogs and eliciting mucosal immune responses in the nose. MDA is also a simpler method of administration in rodents. Moreover, a review comparing administration in humans suggested that oral/gastrointestinal administration induced a lower immune response in the respiratory tract than administration though the sublingual route, whereas the efficacy of buccal administration remains debatable [36].

One important aspect of vaccine efficacy in dogs is the decrease in bacterial load and the shedding of *B. bronchiseptica*. Both oral and intranasal vaccinations in dogs reduced bacterial load in either the trachea or nasal swabs for up to one year after vaccination [17,18]. Consistently with these findings, in our study, the challenge strain was not detected in the nose, trachea, or lungs for up to two weeks after challenge in vaccinated rats (Figure 2A). Moreover, other *Bordetella* vaccine studies in rodents also measured respiratory tract (lung, trachea, and nasal lavages) colonization after challenge and reported similar results [20]. In contrast, certain published literature reports a lack of bacterial load reduction in dogs [12,14]. However, these studies only performed undifferentiated bacterial culture for detection, and it was done shortly after vaccination. This difference may therefore be due to a failure of these studies to distinguish between the challenge and vaccine strains. Indeed, in a study in which nasal swabs were collected after vaccination and before challenge, positive results for bacterial isolation were obtained for animals receiving vaccines via the mucosal route [16]. One of the major strengths of our study was the availability of primers specifically targeting the challenge and vaccine strains, allowing the differentiation between these two strains via qPCR. The bacterial load of the challenge strain attributed to each animal cannot be confused with the persistence of the vaccine strain in the respiratory tract. The use of these specific primers enabled the revelation that vaccine strain persistence was similar in the nose, trachea, and lungs 28 days after vaccination by both the oral and intranasal route. It has been suggested that the propagation of the vaccine strain in the respiratory tract can stimulate the induction of mucosal IgA [32], which is consistent with our findings. Indeed, there was an insignificant statistical trend towards a positive correlation between vaccine strain detection in the respiratory tract and the induction of IgA in the nose. Moreover, it was observed that antibody levels in the serum and nose were negatively correlated with trachea colonization of the challenge strain following challenge.

The main clinical sign of CIRDC in dogs is coughing, so an ability to decrease coughing is one of the requirements of any canine vaccine against *B. bronchiseptica*. Both the oral and intranasal routes of vaccination decrease disease symptoms in dogs [12]. Cough has been studied in rats since 1939 [24] and more recent studies have focused on cough as a lead symptom for studying the pathogenicity of either *B. bronchiseptica* [21] or *B. pertussis* [22,23]. Nakamura et al. studied the *B. bronchiseptica* factors underlying the production of a cough in rats [21]. Rats were infected with *B. bronchiseptica* and the induced cough was evaluated, together with bacterial load. The number of coughs were counted and the bacterial load in the unvaccinated animals in the study described here were consistent with our findings.

On the other hand, vaccination against *B. pertussis* via both the oral and intranasal routes decreases the number of coughs after challenge [22]. Surprisingly, as described here, coughing was reduced significantly only in intranasally vaccinated rats. However, in studies on dogs, cases were defined based on a spontaneous cough for two or more consecutive days [12]. One limitation of this study was that coughing was measured on separate days, from day 6 post-challenge. Measurements were not taken on consecutive days, so it is possible that some animals did not cough on consecutive days and therefore were not considered to have the disease. Another key consideration is that the mechanisms underlying the cough may differ between dogs and rats [37], and this aspect warrants further exploration. In short, we observed similar immune responses, reductions in bacterial load, similar vaccine replication trends in the respiratory tract via oral or intranasal vaccination, with a varying frequency of coughing. One possible explanation for this could be the presence of the challenge strain in the respiratory tract of the orally vaccinated rats before the first day of sampling after challenge (7 days p.c.). It can be hypothesized that this might have led to the induction of lesions in the respiratory tract such that the cough persisted even though the bacteria were no longer present. However, analyses of the lesions in the respiratory tract demonstrated only that the two routes of vaccination led to a decrease in the severity of respiratory tract lesions two weeks after challenge when compared with that in the nonvaccinated control.

Protective immune responses against *Bordetella* species are characterized by antibodies and Th17 cellular responses [20,38]. However, to our knowledge, no study of the cellular responses induced via vaccination against *B. bronchiseptica* in dogs has ever been published. To gather more insights into the cellular immune responses, immune responses in the spleen, a major organized secondary lymphoid organ, was analyzed. We observed that the ratio of CD3+CD4+ (T helper cells) to CD3+CD8+ (cytotoxic T-cells) cells was similar in the three groups, but that the number of CD3+CD4-CD8-, double-negative (DN) T-cells was surprisingly higher in orally vaccinated animals than in the other groups, suggesting that vaccination could have an influence on their abundance. While there are no known studies directly comparing the percentage of double-negative (DN) T-cells in rat spleens after vaccination, the percentage of CD4 and CD8 out of the total splenocytes has been previously described after *B. pertussis* vaccination in mice [38] and in aging rats [39], and our results are comparable. Additionally, while not significant, the percentage of these populations seems to be reduced by vaccination or challenge, as seen after *B. pertussis* vaccination and challenge in mice [38].

Double-negative T-cells have been identified as important cells for both tolerance and pathology [39]. Among them, we can find natural-killer (NK)T-cells, inflammatory or regulatory DNT, DN gamma-delta T-cells (γδ T-cells) and mucosal-associated T (MAIT)-cells [40]. A role of MAIT-cells could be implicated, based on their known involvement in the defense of the lung and the oral mucosa, and in infectious disease [40]. However, additional markers will be required to differentiate between these populations and determine if they play a pathological or tolerogenic role in this case. Therefore, it is not yet possible to draw firm conclusions. In addition, the levels of inflammatory T-cells, CD3+CD4+IL17+ (Th17) and regulatory CD3+CD4+FOXP3+CD25+ (Treg) T-cells were assessed. IL-17-producing T helper (Th17) cells are a subset of effector T-cells characterized by the induction of inflammatory cytokines essential for some vaccine responses but also associated with autoimmune diseases [41]. For instance, respiratory syncytial virus (RSV) infection is more severe if the Th17 response is excessive, but regulatory T-cells can help to counteract the inflammation, preventing disease exacerbation [42]. Additionally, an imbalance of Th17:Treg cells has been observed to be associated with the severe inflammation seen in COVID-19 [43], with acute coronary syndromes (ACS) [44], or the pathogenesis of *Porphyromonas gingivalis* [45]. Hence, an imbalance in the proportions of inflammatory (Th17) and regulatory T-cells (Treg) might account for the clinical signs observed in this study. During data analysis, it was observed that the Th17:Treg ratio 7 days p.c. was significantly higher in orally vaccinated rats when compared with that in intranasally vaccinated rats. However, these data should be interpreted with caution because other mechanisms and cell populations may be at play in modulating these immune responses. Moreover, investigations of the local immune responses in the lung are required to confirm or refute this hypothesis. Differences in cellular responses to oral or nasal administration were previously observed in mice [46]. Sudo et al. showed that the oral and nasal administration of bacterium-like particles (BLPs) conjugated to an antigen led to a differential secretion of cytokines in lymphoid tissues, such as the spleen, nasal-associated lymphoid tissue (NALT) or Peyer’s patches (PPs) [46]. This evidence highlights the relevance of the route of administration based on each target, animal species or desired immune response.

The rat model is a valuable tool for the study of clinical symptoms, bacterial load, and humoral and cellular immune responses to *B. bronchiseptica*. In addition, the reagents available for immunological studies in rats are superior to those available for canine studies, and rats are more easily accessible than dogs are for research studies. Rats and dogs also have several important anatomic and immunological features in common. A study comparing oral mucosae between species, performed by Thirion-Delalande et al., showed similarities in the immune cell populations present in the oral cavities of rats and dogs [47]. Moreover, the naval cavity of the rat is similar anatomically to that of the dog, both these cavities being much less similar to the human nasal cavity [48]. However, there are also major differences in immunological structures between these two species. For example, the rat spleen is considered to be involved in immune defenses, whereas the canine spleen specializes in blood storage and filtration and has underdeveloped immune structures [49]. There are also differences in the organization and structure of gut-associated lymphoid tissue (GALT) and the nose-associated lymphoid tissue (NALT) between these two species [49]. For example, tonsils, which play a key role in host defense and antibody responses to oral and nasal pathogens [50], are present in dogs but not in rats [51]. In our study, the vaccine was administered via the nose and the mouth. It is therefore possible that the differences in NALT organization between dogs and rats can account for the differences in clinical signs between these two species.

Importantly, more studies are required to characterize the cellular immune response against *B. bronchiseptica* more fully. Furthermore, the organs involved in these responses are not only in the spleen but also in the local tissues of the respiratory tract and thus should be further investigated. Both the intranasal and oral vaccination routes have been shown to elicit protection for more than a year in dogs [17,18]. An understanding of the mechanisms involved will be required for the development of the next generation of vaccines. Finally, assessments of antibody functionality will also be required to evaluate the correlation between the presence of antibodies and disease correctly.

Notwithstanding these limitations, our findings suggest that mucosal vaccination against *B. bronchiseptica* provides effective protection against colonization and shedding of the challenge strain in rats. The reasons for the differences in clinical signs observed remain unclear, but oral vaccine administration remains more convenient than nasal administration, which can be difficult in dogs. This study constitutes a first step towards enhancing the understanding of the immune response to *B. bronchiseptica* and the immunological differences between routes of administration. It also highlights the rat model as a surrogate system suitable for use in the future development of mucosal vaccines for dogs. *B. bronchiseptica* is the major pathogen responsible for CIRDC, but it is not the only pathogen implicated in this disease [52]. This model could be used to assess protection against other respiratory pathogens involved, such as canine parainfluenza virus (CPIV). Rats are naturally susceptible to the Sendai virus, which is of the same family as CPIV, the Paramyxoviridae family [53]. Therefore, in future studies, it would be of interest to demonstrate the susceptibility and induction of the disease of CPIV in rats. Thus, rats could potentially be used as a surrogate model for preliminary in vivo testing before conducting definitive tests in dogs.

## Figures and Tables

**Table 1:** Forward and reverse primers used to detect the used challenge and vaccine strains of *B. bronchiseptica*.

Target	Forward Primer	Reverse Primer	FAM
Challenge strain	5′ GCGCAGATGCAGGAAATC 3′	5′ GGCATACAGCGGATAGAG 3′	5′ FAM ccacAcccAtgCacgt BHQ1 3′
Vaccine strain	5′ GGGAACCAAGAACAAGAA 3′	5′ GGATGTAGAGCGAAATAGG 3′	5′ FAM-GCAAGGACAAGGAAGCCAACTG -BHQ1

**Table 2:** Antibodies used to identify T-cell populations upon employing flow cytometry.

Cell Marker	Fluorophore	Staining	Volume in 50µL of Buffer	Supplier	Catalog Number
CD3	FITC	Surface	1 µL	BD	557354
CD4	Alexa Fluor 700	Surface	2.5 µL	BIO RAD	MCA55A700
CD8	PerCP	Surface	2.5 µL	BD	558824
CD25	BV421	Surface	1 µL	BD	565608
FOXP3	PE	Intracellular	2.5 µL	BioLegend	320008
IL-17	APC	Intracellular	1 µL	EBioscience	17-7177-81
Viability	LiveDead Yellow	-	200 µL 1/1000 diluted	Invitrogen	L34959

## Data Availability

Data are available upon reasonable request.

## Supplementary Materials

The following supporting information can be downloaded at: https://www.mdpi.com/article/10.3390/vaccines11050982/s1;. Table S1: Histological rapport of lung and trachea after challenge; Figure S1: Hemolytic activity of the vaccine and challenge strain; Figure S2: Binding strength (avidity) of anti-Bb vaccine IgA in nasal lavages after challenge; Figure S3: Detection of fecal and serum anti-Bb vaccine IgA; Figure S4: Bacterial load of the challenge strain in the unvaccinated group after challenge; Figure S5: Signs of inflammation in the lung and trachea after challenge; Figure S6: Gating strategy flow cytometry; Figure S7: Treg and Th17 populations determined via flow cytometry.
