# Supplementary material for: Mucosal Vaccination with Live Attenuated Bordetella bronchiseptica Protects against Challenge in Wistar Rats
Source: Vaccines (Basel). 2023 May 15;11(5):982. doi: 10.3390/vaccines11050982 (PMC10224215; doi:10.3390/vaccines11050982)
Supplement: Supplementary file 1 [file vaccines-11-00982-s001.zip › vaccines-2366689-supplementary.pdf]

Table S1: Histology rapport of Lung and Trachea after challenge. Histology of the lesions observed in the trachea and lung 7- and 14- days after challenge per group and per day after challenge. (n=4 per group per day).

I. Summary of the main microscopic findings

Group		Oral vaccination		Nasal vaccination		Control	
Day of sampling		D7	D14	D7	D14	D7	D14
Number of animals		4	4	4	4	4	4
**Lung**							
Inflammation, bronchioloalveolar, multifocal to coalescing							
	Minimal	3	2	2	1	3	1
	Mild	-	2	-	3	-	3
Bronchus, inflammation, focal/multifocal							
	Minimal	-	1	-	1	-	2
	Moderate	-	-	-	-	-	1
Lymphoid follicles							
	Minimal	1	1	1	1	-	1
	Mild	-	-	-	-	-	1
Bronchus/bronchioles : presence of bacteria		-	-	-	-	-	-
**Trachea**							
Mononuclear inflammation, diffuse							
	Minimal	2	-	3	1	-	2
	Mild	-	-	-	-	-	1
Epithelial alteration							
	Minimal	-	-	2	-	-	1
Presence of bacteria		-	-	-	-	-	-

- no changes

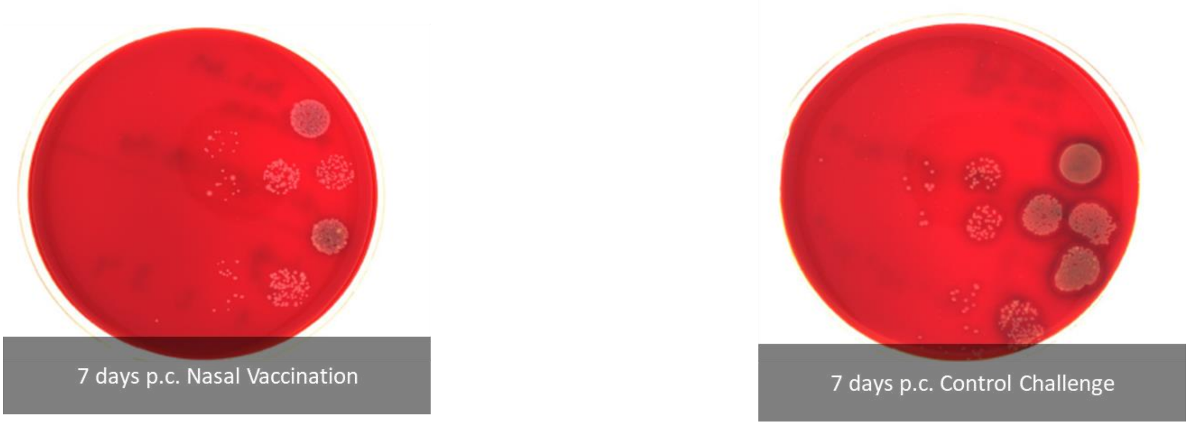

Figure S1: Hemolytic activity of the vaccine and challenge strain. The vaccine and challenge strains were cultured in Bordet Gengou agar containing 15% sheep blood at 37°C for 48 hours, and the number of colony-forming units (CFUs) was determined. The challenge strain's hemolytic activity allowed for visual differentiation of both strains, as the vaccine strain lacked hemolytic activity. The definitive confirmation was done using qPCR

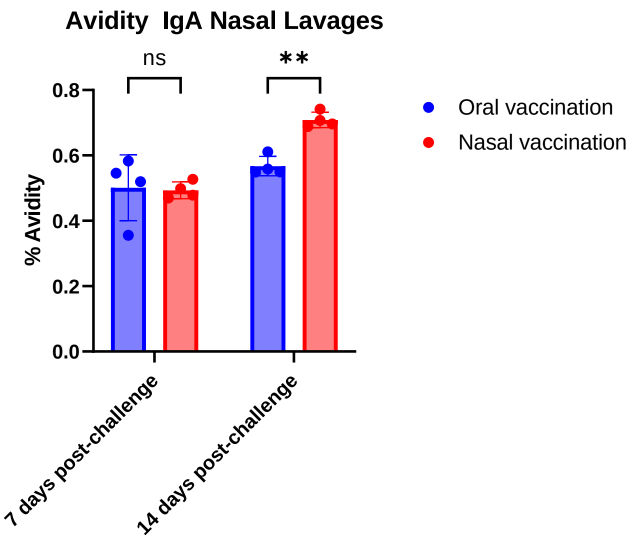

Figure S2: Binding strength (avidity) of anti-Bb vaccine IgA in nasal lavages after challenge. Nasal lavages were collected 13 post-boost, 7- and 14-days post-challenge. Anti-Bb vaccine IgA were measured with and without an incubation with10min with 6M of urea and the avidity index was determined by the ratio of both measured OD (% Avidity). The results are shown as % of Avidity and mean with SD are presented. Each dot represents an individual rat. (n=4 per group per day). P values were determined by two-way ANOVA (** p ≤ 0.01).

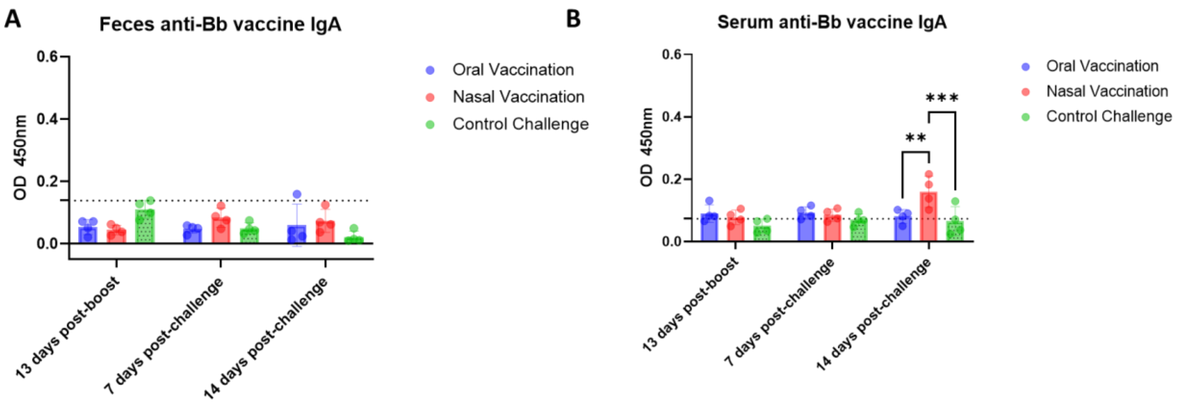

Figure S3: Detection of fecal and serum anti-Bb vaccine IgA. Blood was collected at euthanasia by the intracardiac route 13 days post-boost and 7- and 14- days post-challenge. Feces were also collected at the same timepoint. Anti-Bb vaccine IgA were measured in feces and serum and differences in the levels of the groups were compared at each timepoints. (**A**) Anti-Bb vaccine IgA levels in feces were low before and after challenge, with no significant differences observed between groups. (**B**) Before challenge, all groups showed low levels of anti-Bb vaccine IgA in serum. However, 14 days post-challenge, the nasal vaccinated group showed a significant but low increase in IgA levels compared to the other groups. The results are shown with the DO and mean with SD are presented. Each dot represents an individual rat. (n=4 per group per day). P values were determined by two-way ANOVA (** p ≤ 0.01; *** *p* ≤ 0.001)

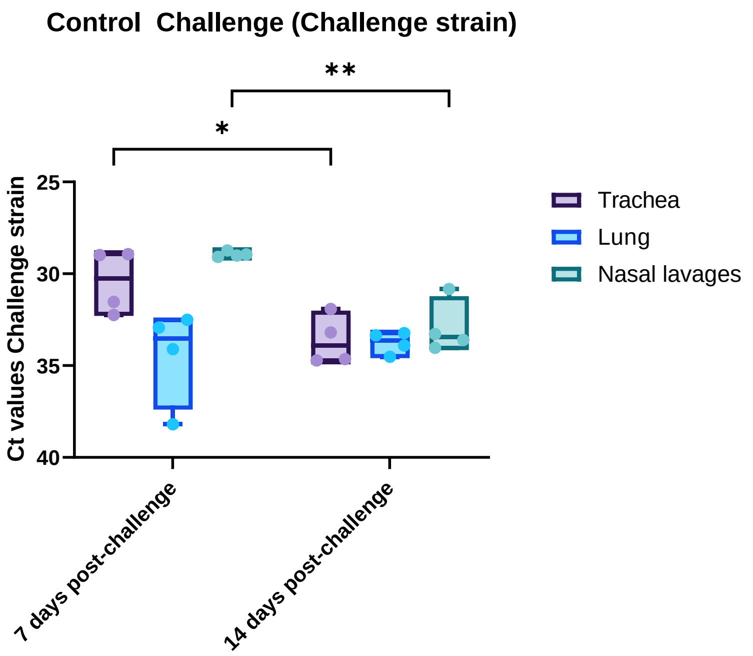

Figure S4: Bacterial load of the challenge strain in the unvaccinated group after challenge. 7 and 14 days after challenge, the presence of the challenge and vaccine strain was determined by qPCR in the lung, trachea, and nasal lavages of euthanized rats. The control challenged group showed the presence of the challenge strain in the respiratory tract, confirming a successful challenge. The bacterial load 7 days post-challenge was higher in the nasal lavages and trachea of the control group compared to the lungs. There was a significant decrease in bacterial load 14 days post-challenge compared to 7 days post-challenge in the trachea and nasal lavages. Each dot represents an individual rat and the error bars represent the standard error of the mean (n=4 per group per day). Statistical significance determined by ANOVA with multiple comparisons is denoted by asterisks and brackets as follows: * $p \leq 0.05$; ** $p \leq 0.01$

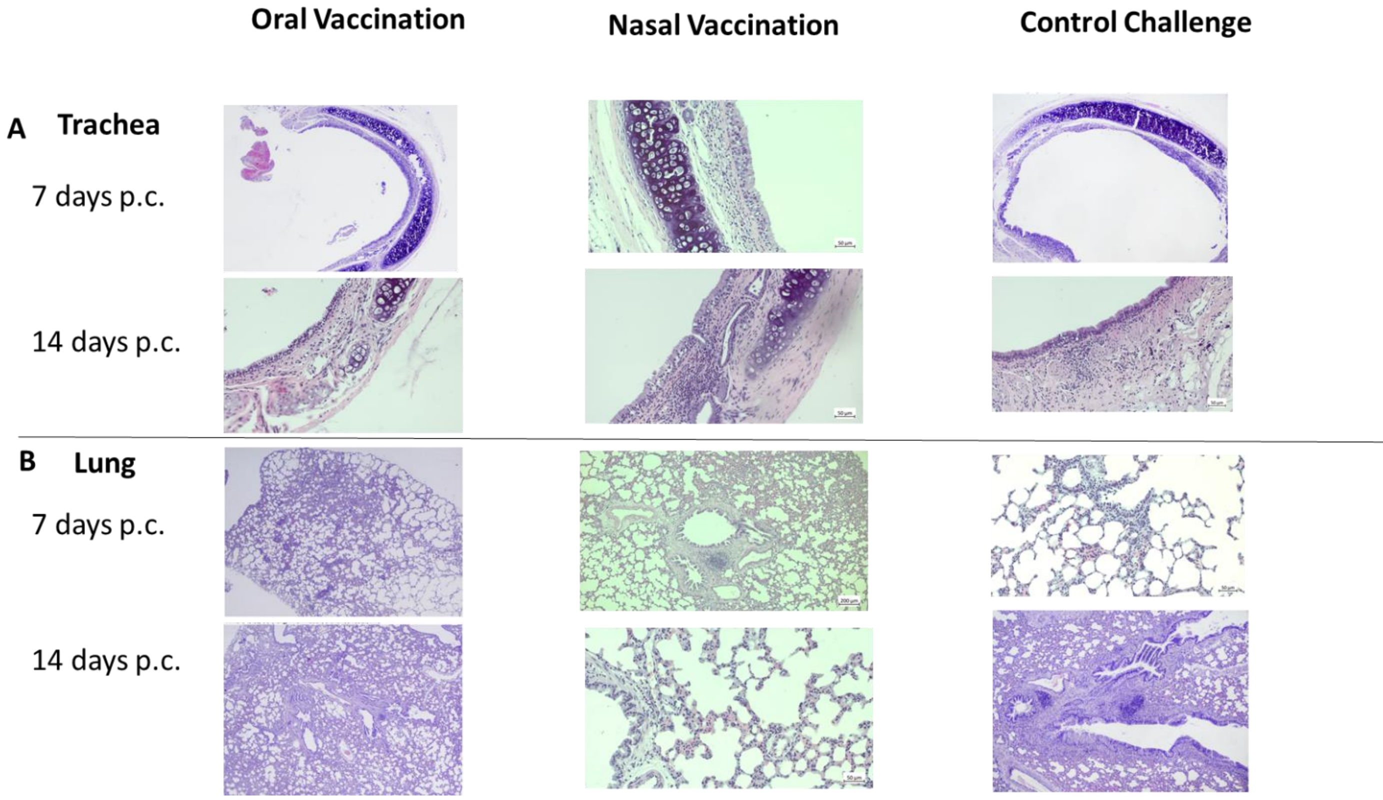

Figure S5. Signs of inflammation in the lung and trachea after-challenge. After challenge, the lung and trachea of each animal were stained with Hematoxylin and Eosin (H&E), and images were taken at 5X or 20X magnification. (A) Lesions in the trachea of vaccinated and unvaccinated animals 7 and 14 days after challenge showed higher inflammation in the unvaccinated group at day 7. (B) Lesions in the lung of vaccinated and unvaccinated animals 7 and 14 days after challenge showed higher inflammation in the unvaccinated group at day 14. (n=4 per group per day).

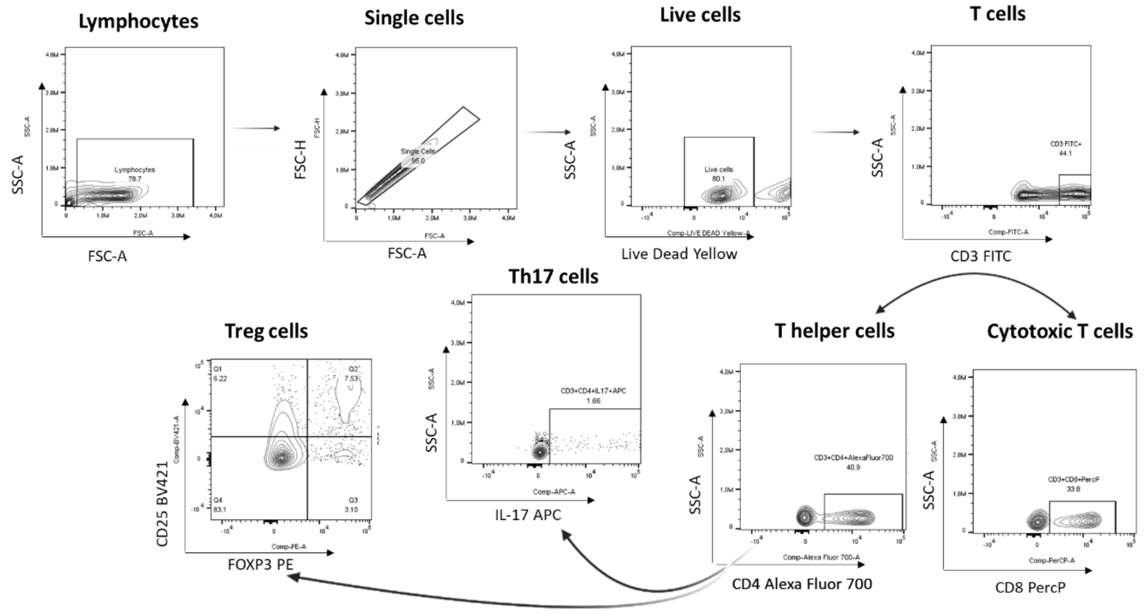

Figure S6: Gating strategy flow cytometry. The gating strategy for flow cytometry involved selecting lymphocytes from the total cells, followed by gating for single and live cells. T cells were identified as CD3+ cells, and T helper cells (CD3+CD4+) and cytotoxic T cells (CD3+CD8+) were separated. Within the T helper cell population, Th17 cells (CD3+CD4+IL17+) and Treg cells (CD3+CD4+FOXP3+CD25+) were identified.

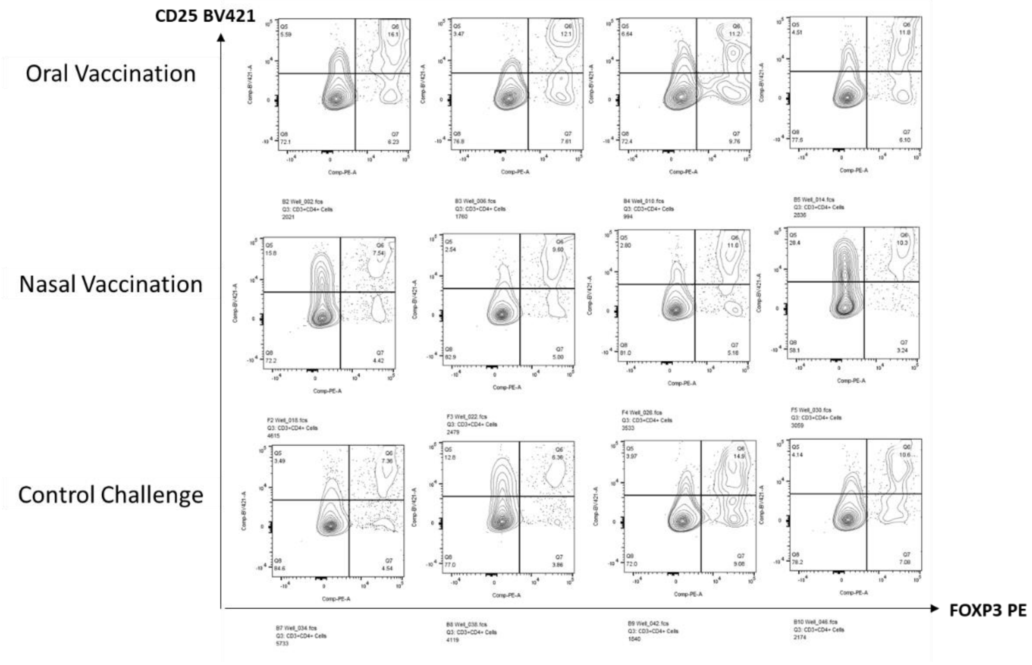

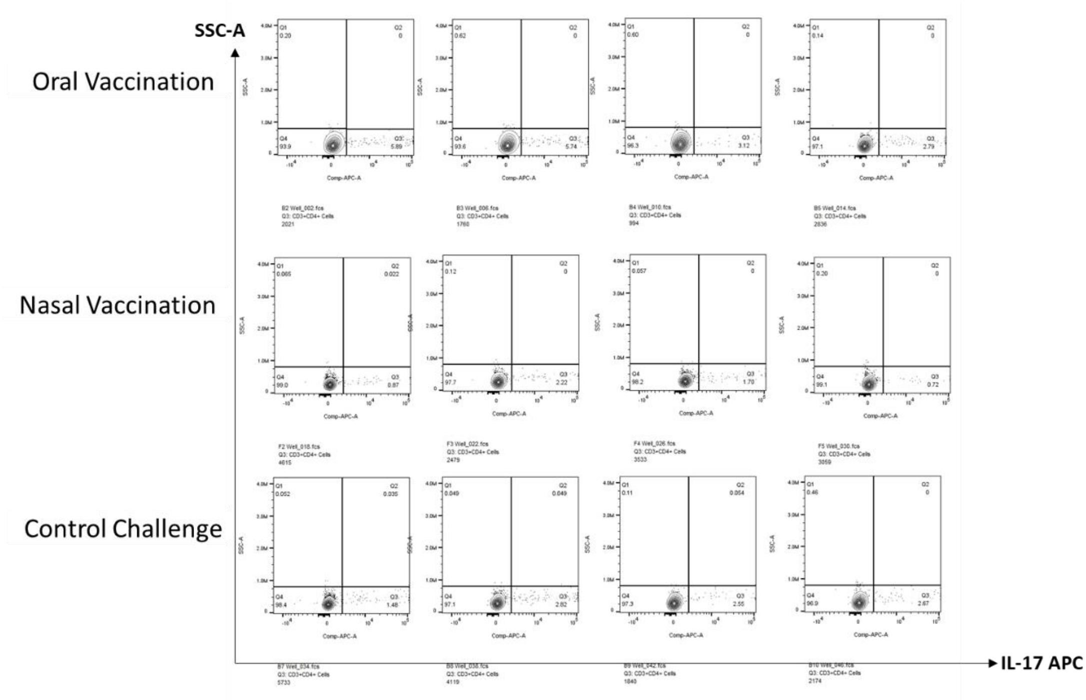

Figure S7: Treg and Th17 populations by flow cytometry. For each group of animals 7 days post-challenge, flow cytometry dot plots were generated to gate the following cell populations based on their markers: CD3+CD4+FOXP3+CD25+ (Treg cells), CD3+CD4+FOXP3+CD25-, CD3+CD4+FOXP3-CD25+, and CD3+CD4+FOXP3-CD25-. (n = 4 per group) (**A**). Similarly, for Th17, the cell populations were identify based on the markers: CD3+CD4+IL17+ (Th17), and CD3+CD4+IL17- (Other T helper cells). (n = 4 per group) (**B**).
